# Review of mathematical models of HSV-2 vaccination: Implications for vaccine development

**DOI:** 10.1016/j.vaccine.2018.02.067

**Published:** 2019-11-28

**Authors:** Ian H. Spicknall, Katharine J. Looker, Sami L. Gottlieb, Harrell W. Chesson, Joshua T. Schiffer, Jocelyn Elmes, Marie-Claude Boily

**Affiliations:** a Division of STD Prevention, Centers for Disease Control and Prevention (CDC), Atlanta, GA, USA; b Population Health Sciences, Bristol Medical School, University of Bristol, Bristol, UK; c Department of Reproductive Health and Research, World Health Organization (WHO), Geneva, Switzerland; d University of Washington, Seattle, WA, USA; e Fred Hutchinson Cancer Research Center, Seattle, WA, USA; f Department of Infectious Diseases Epidemiology, Imperial College London, UK

**Keywords:** Mathematical models, Vaccine impact, Herpes simplex

## Abstract

Development of a vaccine against herpes simplex virus type 2 (HSV-2), a life-long sexually-transmitted infection (STI), would be a major step forward in improving global sexual and reproductive health. In this review, we identified published literature of dynamic mathematical models assessing the impact of either prophylactic or therapeutic HSV-2 vaccination at the population level. We compared each study’s model structure and assumptions as well as predicted vaccination impact. We examined possible causes of heterogeneity across model predictions, key gaps, and the implications of these findings for future modelling efforts. Only eight modelling studies have assessed the potential public health impact of HSV-2 vaccination, with the majority focusing on impact of prophylactic vaccines. The studies showed that even an imperfect prophylactic HSV-2 vaccine could have an important public health impact on HSV-2 incidence, and could also impact HIV indirectly in high HIV prevalence settings. Therapeutic vaccines also may provide public health benefits, though they have been explored less extensively. However, there was substantial variation in predicted population-level impact for both types of vaccine, reflecting differences in assumptions between model scenarios. Importantly, many models did not account for heterogeneity in infection rates such as by age, sex and sexual activity. Future modelling work to inform decisions on HSV vaccine development and implementation should consider cost-effectiveness, account for additional HSV-2 sequelae such as neonatal transmission, and model greater heterogeneity in infection rates between individuals, more realistic vaccine deployment, and more thorough sensitivity and uncertainty analyses.

## 1 Introduction

Development of a vaccine against herpes simplex virus type 2 (HSV-2), a life-long sexually-transmitted infection (STI), would critically improve global sexual and reproductive health. In 2012, 417 million people aged 15–49 years were estimated to be infected with HSV-2, 64% of whom were women [1]. HSV-2 infection can cause recurrent painful genital lesions and is often associated with negative psychosocial effects such as shame, anxiety, and depression [2], [3]. Although neonatal herpes from mother-to-child transmission is rare, it has high morbidity and mortality [4]. In addition, evidence suggests that HSV-2 infection increases susceptibility to and infectivity with HIV [5], [6], [7]. This increased HIV susceptibility is likely due to increases in activated CD4+ HIV target cells and breaks in the genital mucosa during active HSV infection, which facilitate viral entry. HSV-2 infection correlates with higher levels of HIV viraemia and therefore infectivity [8], [9], [10], [11]. Herpes simplex virus type 1 (HSV-1) is related to HSV-2 and is also a common lifelong viral infection. Although HSV-1 is usually acquired orally during childhood, causing orolabial clinical manifestations [12], it can also be acquired genitally, causing genital ulcers. Genital HSV-1 infection is usually milder than HSV-2, causing less frequent recurrences of symptomatic ulceration and viral shedding [13], but neonatal transmission risk may be higher for HSV-1 than HSV-2 if shedding occurs during delivery [14]. Evidence suggests that previous HSV-1 infection reduces the risk of symptomatic HSV-2 seroconversion [15].

Antiviral treatment with medications such as acyclovir, famciclovir, and valacyclovir can reduce severity and frequency of HSV genital symptoms [16], and a large trial in high-income countries showed daily valacyclovir can reduce HSV transmission to sex partners by 48% [17]. However, treatment is a sub-optimal prevention tool since it cannot cure infection and has incomplete impact on infectivity, even when taken daily [18]. Though past vaccine candidates have not achieved desired clinical efficacies [19], [20], advances in adjuvants, our understanding of HSV immunology, and development of efficacious vaccines against varicella zoster virus, a closely related alpha-herpesvirus, have led to renewed interest in HSV vaccine development [21]. Several novel HSV vaccine candidates are now in clinical and pre-clinical development, holding promise for addressing the public health burden of genital herpes [21], [22]. A global roadmap recently outlined critical steps to facilitate and accelerate STI vaccine development and decision-making [23]. One of these key steps is to model the potential impact (i.e., population-level effectiveness and cost-effectiveness) of vaccination against HSV-2.

Transmission dynamic models (i.e., dynamical models, here onward) are increasingly used for informing vaccination policies because they include herd effects, which are needed to evaluate the population-level impact across multiple vaccination strategies. Dynamical models can be used at different stages of vaccine development [24], [25], [26], [27], [28]. Early on, dynamical models can inform investment cases for vaccine development and guide decision-making by vaccine developers and funders. Dynamical modeling can also help determine characteristics a vaccine must have in different settings to maximize improvements in population health. As vaccine development progresses and safety and efficacy data accumulate from clinical trials, model-based impact analyses can inform policy decisions on whether and how to optimally introduce vaccination in different settings, e.g., clarifying target populations and implementation strategies given the specific characteristics of the vaccine and population. Models are also valuable to inform the design and interpretation of surveillance studies post-vaccination [29], [30], [31].

In 2015 the World Health Organization (WHO) hosted an expert consultation on HSV-2 vaccine impact modeling [32] to understand what modeling work had already been done, remaining gaps, and questions and key considerations for future models in order to inform an investment case for HSV-2 vaccine development and preferred product characteristics. In preparing for the consultation, and subsequently to catalyze new modeling efforts to inform HSV-2 vaccine development, we reviewed the published literature on mathematical models assessing population-level effectiveness of HSV-2 vaccination. The objectives of the review were to describe the models’ structures and assumptions, summarize predicted vaccination impact, discuss possible causes of heterogeneity across model predictions and explore the implications of these findings for future modeling efforts.

## 2 Methods

We conducted a literature review to identify peer-reviewed publications of dynamical models of population-level impact of HSV-2 vaccination. We searched PubMed for literature published between 1 January 1980 and March 2017, using the following untagged search terms: ‘(vaccine OR vaccination) AND (HSV OR herpes simplex) AND (mathematical model OR simulation model OR individual based model OR agent-based model) NOT (animal).’ We included publications that reported results from dynamical models of HSV-2 vaccination. We excluded publications that solely used (1) within-host models, (2) static/Markov cohort models, (3) commentary or review of prior modeling results, or (4) models that studied HSV-2 transmission dynamics without an intervention, or only studied other non-vaccine interventions (e.g. HSV-2 antiviral treatment). The abstract and title of records retrieved from the search were scanned for relevance. The full texts of potentially-relevant publications were then reviewed to confirm relevance. References of included publications were perused to identify additional relevant publications.

We extracted information describing the key features and assumptions of each modeling study on: (i) population (demography, sexual behavior, setting) and calibration, (ii) HSV-2 natural history, (iii) vaccine characteristics and vaccination strategy, (iv) health outcomes modeled to predict impact (e.g., HSV-2 infection, HIV infection, neonatal infection, economic outcomes) [28]. We compared predicted epidemiological impact of vaccination using the most commonly-reported outcome across models (i.e. HSV-2 incidence), which was extracted from the main text, tables, and figures. Since one study, Newton et al. [33], only presented results on HSV-2 prevalence, and the model was easy to reproduce, we recoded it using the same assumptions and parameter values to derive vaccination impact on HSV-2 incidence for the same scenarios explored in the publication (details of these methods are in Supplemental materials).

## 3 Results

### 3.1 Search results

Of the 45 potentially-relevant modeling publications identified, seven met the inclusion criteria (details in Supplemental Fig. S1). One additional publication [34] was identified from the reference lists of included publications [33], [35], [36], [37], [38], [39], [40]. In total, eight unique dynamical modeling studies, published between 2000 and 2012, that assessed the potential population-level impact of HSV-2 vaccination, were included in our review [33], [34], [35], [36], [37], [38], [39], [40].

### 3.2 Description of model characteristics

**Fig. 1:**
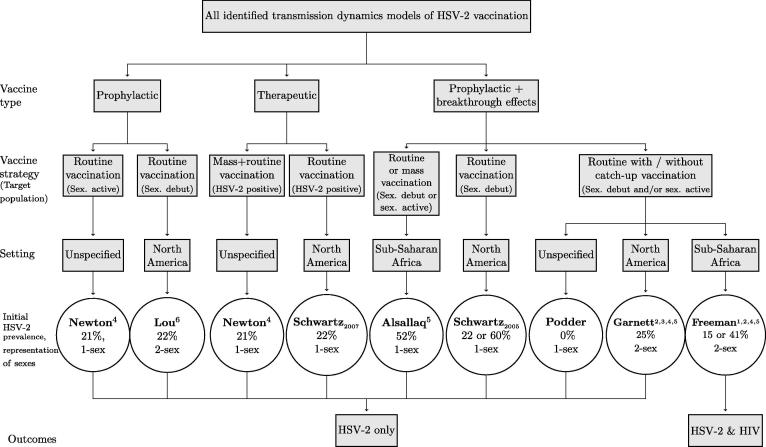
Summary of key model characteristics related to type of vaccine, vaccination strategy, target population, setting and outcomes. Additional details found in Supplemental Table S1, Fig. 2, Fig. 3. The prophylactic and therapeutic vaccine studies focus vaccination on HSV-2 negative and positive people, respectively. 1-sex models combine males and females; 2-sex models represent females and males separately, allowing for vaccination of one or both sexes. A sexually active target population refers to vaccination of people who are already having sex; a sexual debut target population refers to vaccination of people before they become sexually active.

**Fig. 2:**
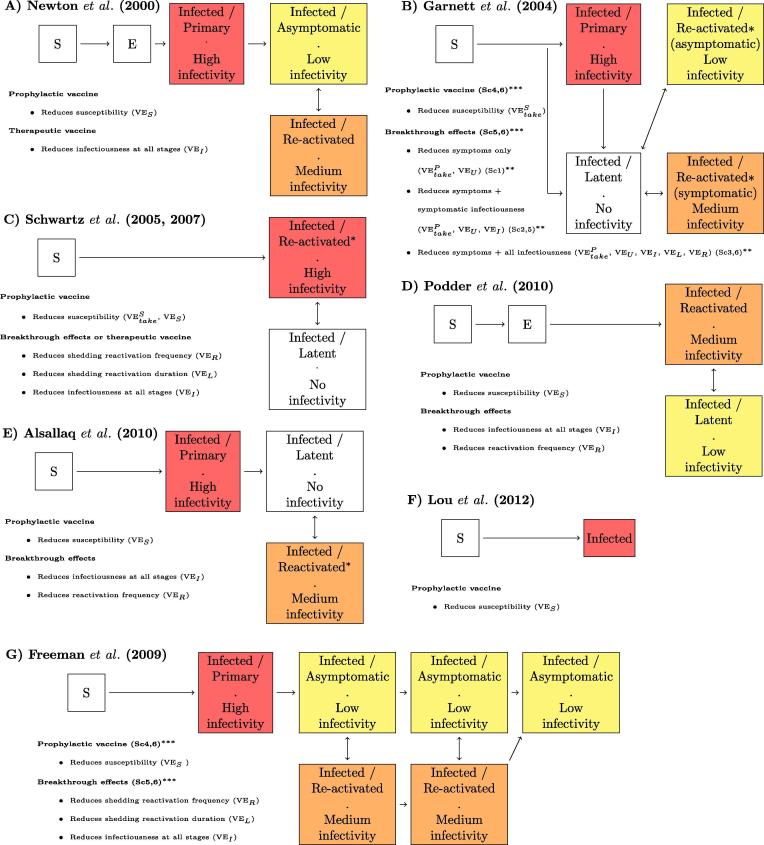
Flowchart of HSV-2 natural history, vaccine effects, and efficacy assumptions in each model. Only Lou et al. (2012) in (F) does not represent reactivation episodes of high infectivity through the life-course of HSV-2 infection. Otherwise, all other models represent reactivation periods associated with increased viral shedding and infectivity and asymptomatic or latent periods associated with low or no infectivity. Panels (B) and (G) refer to specific scenarios defined in the original publications and presented in Figs. 4^**^ and 5^***^. Red, orange, and yellow reflect high, medium and low infectivity within a given model. In addition, Garnett et al. (2004) and Freeman et al. (2009) assume that reactivation frequency declines with time since infection. S – Susceptible; E – Exposed, not infectious. (For interpretation of the references to colour in this figure legend, the reader is referred to the web version of this article.)

Table 1 defines the terminology used to describe vaccine characteristics and vaccination roll-out strategies. Table 2, Fig. 1, Fig. 2, and Supplemental Table S1 summarize the characteristics of the different modeling studies. Seven studies used deterministic compartmental models and one used a stochastic individual-based model [38].

(i) Population and calibration

**Table 1:** Glossary of vaccine and vaccination terms used to describe models (based on Boily et al. (2012)[28]).

Term	Definition
*Vaccine types*	
Prophylactic vaccine	A vaccine given before acquiring infection, primarily intended to prevent infection of the vaccinated host. Some prophylactic vaccines may also reduce disease manifestations and infectivity during *breakthrough infections*, i.e., those occurring despite vaccination.
Therapeutic vaccine	A vaccine given after acquiring infection, primarily intended to improve disease outcomes (progression, severity, occurrence), akin to treatment effects. Therapeutic vaccines may also reduce infectivity.

*Vaccine characteristics and vaccine efficacies (VE)*	
Take – effectively vaccinated	The probability that a vaccinated person will develop an adequate immune response (i.e., be *effectively vaccinated*) that can protect them fully (perfect sterilizing immunity) or partially against infection or disease.
Susceptibility effects –reduction in susceptibility (prophylactic vaccine)	VE^S^*_take_*: Take for a prophylactic vaccine –% effectively protected against infection. VE*_S_*: The percentage reduction in the risk of infection upon exposure among people effectively vaccinated. For example, VE*_S_* = 100% or 50% means that an individual effectively vaccinated (i.e., among whom the vaccine “takes”) has 0% or 50% chance of becoming infected per exposure, respectively.
Breakthrough effects (prophylactic vaccine)	*Infectivity and/or pathogenicity effects* (described below) conferred by a prophylactic vaccine should a vaccinated host subsequently become infected. Not all prophylactic vaccines confer breakthrough effects.
Pathogenicity effects – reduction in disease progression or severity or adverse events (prophylactic or therapeutic vaccines)	VE^P^*_take_*: Take for a therapeutic vaccine –% effectively protected against pathogenicity. VE*_R:_* Percentage reduction in the frequency of symptomatic and/or asymptomatic reactivation (shedding) in effectively vaccinated infected individuals. VE*_L_:* Percentage reduction in the duration of symptomatic and/or asymptomatic reactivation (shedding) in effectively vaccinated infected individuals. VE*_U_:* Percentage reduction in the frequency or duration of ulcerative disease only (no shedding reduction). *Note*: Decreased frequency or duration of reactivation may indirectly reduce transmission by reducing the number of days with viral shedding, viral load, or ulcers, depending on the relationship between disease, shedding and transmission.
Infectivity effects – reduction in infectivity (prophylactic or therapeutic vaccine)	VE^I^*_take_*: Take for a therapeutic vaccine –% effectively protected against infectivity.VE*_I:_* Percentage reduction in the infectivity of effectively vaccinated but infected individuals to their partners, due to a reduction in viral shedding. For example, when VE*_I_* = 100% or 75%, an effectively vaccinated infected individual is not or only 1/4 as infectious as an unvaccinated infected individual, respectively. *Note*: the reduction can be conferred at specific stages or across all stages.
Duration of effects – waning	Lifetime: Efficacy remains constant for the lifetime of the vaccinated individual (i.e., no waning). Finite: Efficacy wanes over time (e.g., after 5, or 20 years), and may necessitate booster vaccination.

*Vaccination roll-out strategies*	
Routine vaccination	Vaccination of a specific target population repeated routinely (e.g., each year).
Catch-up vaccination	Vaccination of individuals who may have been missed or are typically not included in routine vaccination (e.g., older age-cohorts, specific risk groups, gender). Catch-up vaccination campaigns are often implemented for a limited period of time.
Booster vaccination	Vaccination given after an initial vaccination course, to counteract waning vaccine effects. Boosters are most relevant if individuals remain at risk for infection past the period of waning vaccine effects.
Mass vaccination	Vaccination of a large fraction of the population in a very short period of time.
Target population	The population that we aim to vaccinate (e.g., girls, sexually-active adults, pregnant women, newly HSV-2 positive people).
Gender-neutral	Vaccination of both men and women.
Uptake	Fraction of target population vaccinated each year. It may be represented as a rate in models
Coverage	Cumulative fraction of the population that is vaccinated (effectively or not) after a fixed time period; may also be sub-group specific. *Note*: When the *take* is <100%, the coverage and the fraction of individuals effectively vaccinated differ.

*Vaccination scenarios modeled (types of vaccine effects modeled in Garnett* et al. *(2004) and Freeman* et al. *(2009))*	
Sc1	Vaccine affects symptom frequency only (no reduction in susceptibility or infectivity).
Sc2	Vaccine affects symptom frequency and symptomatic infectivity.
Sc3	Vaccine affects symptom frequency and all infectivity (symptomatic and asymptomatic).
Sc4	Vaccine affects susceptibility only.
Sc5	Vaccine affects susceptibility, symptom frequency, and symptomatic shedding/infectivity.
Sc6	Vaccine affects susceptibility, symptom frequency, and all shedding/infectivity.

**Table 2:** Characteristics of the 8 different models (see Fig. 1, Supplement Table S1).

Characteristic	No. of studies	Reference number (s)
*Modelling framework*		
Deterministic compartmental model	7	[33], [34], [35], [36], [37], [39], [40]
Stochastic individual-based model	1	[38]

*Population*		
Sex: No differentiation between the sexes (1-sex)	5	[33], [34], [36], [37], [39]
Males and females represented separately (2-sex)	3	[35], [38], [40]
Duration of sexual activity: Short	4	[33], [36], [37], [40]
Long	6	[34], [35], [36], [37], [38], [39]
Age-structured	1	[38]
Heterogeneous sexual activity classes	3	[35], [38], [39]
Setting: Sub-Saharan Africa	2	[38], [39]
North America	4	[35], [36], [37], [40]
Unspecified	2	[33], [34]
HSV-2 prevalence: Low (15–25%)	6	[34], [35], [36], [37], [38], [40]
High (35–60%)	4	[33], [36], [38], [39]
Model calibration to specific setting: None	1	[34]
1 manual fit	4	[33], [35], [39], [40]
2 manual fits	1	[38]
Sensitivity analysis	2	[36], [37]

*HSV-2 natural history*		
Initial infection explicitly modeled	4	[33], [35], [38], [39]
Recurrent heterogeneous infectivity stages (i.e., reactivation = symptomatic and/or asymptomatic shedding)	7^a^	[33], [34], [35], [36], [37], [38], [39]
Recurrence of symptoms reduces sexual activity	1	[35]
Symptoms/reactivation frequency declines with infection time	2	[35], [38]
HSV-2 treatment or HIV treatment	0	–

*Vaccine characteristics*		
Therapeutic vaccine	2	[33], [37]
Prophylactic vaccine (with or without breakthrough effects)	7	[33], [34], [35], [36], [38], [39], [40]
Duration of vaccine effects: Lifelong	5	[33], [35], [38], [39], [40]
Finite		[34], [36], [37], [38]

*Target population*		
Female-only	3	[35], [38], [40]
Gender neutral	7	[33], [34], [35], [36], [37], [38], [39]
HSV-2 negative	7^b^	[33], [34], [35], [36], [38], [39], [40]
HSV-2 positive	2	[33], [37]
Before sexual debut	6	[34], [35], [36], [38], [39], [40]
After sexual debut	6	[33], [34], [35], [37], [38], [39]

*Vaccination strategies × target population*		
Routine vaccination (with and without catch-up):Before sexual debut - HSV-2 negative	6	[34], [35], [36], [38], [39], [40]
Sexually active - HSV-2 negative	1	[33]
Sexually active - HSV-2 positive	1	[37]
Mass vaccination: HSV-2 negative	1	[39]
HSV-2 positive	1	[33]

*Health outcomes modeled*		
HSV-2 frequency: Incidence rate^c^	3	[35], [38], [39]
Risk^d^	3	[36], [37], [39]
Prevalence	6	[33], [34], [35], [38], [39], [40]
HIV-1 incidence	1	[38]
Health economic outcomes	0	–
Neonatal transmission	0	–

a Only Garnett modeled symptomatic and asymptomatic reactivation separately and a latent (i.e., no shedding) phase.

b Target population in Garnett and Lou also HSV-1 negative.

c After 10 or 30 years.

d Cumulative incidence over ten years.

All models represented open populations (i.e. people entering and leaving the population) that were assumed to be sexually active for various durations ranging from 9 years (representing only young adults [33]) to 35 years (representing younger and older adults [35], [39]). Five models [33], [34], [36], [37], [39] did not distinguish between sexes (i.e., females and males were combined) whereas three models explicitly represented males and females [35], [38], [40] (Fig. 1, Table 2). Only one model was age structured [38], and three models included heterogeneous sexual activity classes [35], [38], [39]. Models either represented settings in sub-Saharan Africa (SSA) [38], [39], North America [35], [36], [37], [40], or unspecified generic settings [33], [34]. Baseline HSV-2 prevalence was assumed to be low only (15–25%) (N = 4) [34], [35], [37], [40], high only (35–60%) (N = 2) [33], [39], or both (N = 2) [36], [38]. Model calibration was minimal. Freeman et al. manually fitted the model to setting-specific demographic characteristics and age-stratified HSV-2 and HIV prevalence, manually identifying one fitting parameter set for each setting (Cotonou, Benin, and Kisumu, Kenya). Schwartz et al. simultaneously varied and identified multiple parameter values reproducing the desired HSV-2 prevalence. One study [34] did not fit to any historical HSV-2 infection pattern. All others manually fitted models to a single HSV-2 prevalence using a single parameter set. Six studies assessed vaccination impact assuming HSV-2 was at or near equilibrium (N = 6) [35], [36], [37], [38], [39], [40] while two assumed HSV-2 was in its initial growth phase (N = 2) [33], [34] although the latter will not be representative of most real-life settings.

(ii) HSV-2 natural history

Fig. 2 illustrates how each model represented HSV-2 natural history at the individual level. During initial or primary HSV-2 infection, individuals can develop genital ulcers and systemic symptoms lasting 2–3 weeks. Individuals typically shed high quantities of virus and are likely to be highly infectious during this phase [41], [42]. During initial infection, HSV-2 establishes chronic infection in the sensory ganglia, where the virus periodically “reactivates”, resulting in viral transport to the genital mucosa and HSV-2 replication, shedding, and infectivity. HSV-2 reactivations can occur with or without accompanying genital lesions. When lesions occur in the presence of replicating genital HSV, this is often referred to as a “recurrence”, “outbreak” or simply “symptomatic shedding”; when the virus is present in the genital tract without accompanying lesions, it is referred to as “asymptomatic shedding” [9]. Typically, viral shedding occurs with a higher viral load during recurrences than asymptomatic shedding [43], [44], but regardless of symptoms, chronic HSV-2 infection alternates between periods of lower and higher infectivity.

In the modeling studies reviewed, the initial phase was often referred to as “primary” infection; subsequent stages of infection with low or no infectivity were referred to as “asymptomatic”, “quiescent” or “latent” periods, and stages with increased viral shedding and higher infectivity as “reactivation” or “recurrence”. The terminology used in these epidemic models sometimes differed from that used in the clinical literature. For example, in the clinical literature, primary infection is sometimes defined as infection with one HSV type among individuals without pre-existing infection with the other type. Moreover, clinically speaking, “asymptomatic” periods may or may not include shedding of virus at levels sufficient for sexual transmission. Nevertheless, the critical feature is how low and high infectivity states were modeled. All but one model [40] represented long periods of low [33], [34], [35], [38] (or even zero [35], [36], [37], [39]) infectivity and shorter periods of higher infectivity due to reactivation (Fig. 2). Two studies reflected that the frequency of symptoms and reactivation decay with time since infection [35], [38]. Only one study assumed that sexual activity was reduced during symptomatic recurrences [35].

HSV-1 has interactions with HSV-2 that may influence HSV-2 transmission dynamics and vaccination impact, e.g., if HSV-2 vaccination also protects against HSV-1, if it protects differentially among people infected with HSV-1, or if HSV-1 alters the natural history of HSV-2 and vice versa. None of the models dynamically modeled co-circulating HSV-1 and HSV-2 infections. However, two studies modeled HSV-1 statically by assuming that a constant population fraction was HSV-1 infected [35], [40], in order to reflect the results of an early vaccine trial showing that vaccination was only effective among HSV-1 negative women [19]. Only one publication modeled co-circulating HSV-2 and HIV infections [38] (Fig. 1, Table 2), assuming that HSV-2 increased susceptibility to and infectivity with HIV during initial HSV-2 infection and reactivation episodes and estimated the number of HIV infections prevented by HSV-2 vaccination. Importantly, none of the models accounted for HSV-2 treatment.

(iii) Vaccine characteristics and vaccination strategy

Vaccines may be broadly categorized as either (i) prophylactic, if they are administered to susceptible individuals primarily to prevent infection (they may also provide “therapeutic” benefits following breakthrough infections), or (ii) therapeutic, if they are administered after infection to “treat” or “modify” infection by alleviating symptoms and/or reducing shedding and infectivity. Only one publication explicitly modeled therapeutic vaccines, with pathogenesis and infectivity effects included [37], though another [33] mimicked a therapeutic vaccine by assuming that when the vaccination program starts all past and future HSV-2 infected individuals immediately have reduced infectivity (akin to mass vaccination of prevalent cases + routine vaccination of all future incident cases) (Fig. 1). Seven publications modeled a prophylactic vaccine, either with (N = 5) [34], [35], [36], [38], [39] or without (N = 2) [33], [40] breakthrough effects reducing subsequent infectivity and/or pathogenicity (Fig. 1, Fig. 2, Table 2, Supplement Table S1). Vaccine effect durations were either finite (5, 10, or 15 years) [34], [36], [37], [38] or lifelong [33], [35], [38], [39], [40] (Table 2). Vaccination roll-out strategies modeled included mass vaccination [33], [39] and routine vaccination with [34], [35], [38] or without [33], [35], [36], [37], [38], [39], [40] catch-up of the target population, which was either individuals before sexual debut [34], [35], [36], [38], [39], [40], already sexually active [33], [34], [35], [37], [38], and HSV-2 negative [33], [34], [35], [36], [38], [39], [40] or HSV-2 positive [33], [37] (Fig. 1, Table 2). All models except one [40] explored vaccination of both sexes and three studies explored female-only vaccination [35], [38], [40].

(iv) Outcomes modeled

Most models reported the population-level effectiveness of vaccination as the relative reduction in HSV-2 incidence rate after 10 or 30 years or in cumulative incidence over 10 years (i.e., infection risk) in the overall population following the start of theoretical vaccination. One [35] reported results for each sex and only one other [38] predicted reductions in HIV incidence following prophylactic HSV-2 vaccination. No publications considered neonatal or any health economic outcomes (Table 2). Because the analyses of two publications [34], [40] primarily focused on eradication thresholds, and mostly made predictions after 250 or 1000 years, they were excluded from our comparisons across models in the next section (Supplemental Table S1).

## 4 Summary of population-level effectiveness predictions of vaccination

**Fig. 3:**
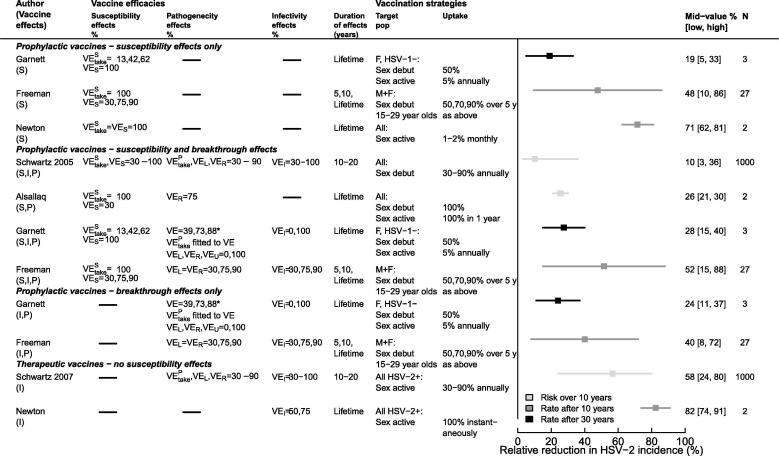
Predicted reduction in HSV-2 incidence rate or risk in the overall population (except Garnett, which reports results by sex) by vaccine types – prophylactic, with or without breakthrough effects, and therapeutic – for the set of vaccine efficacies, duration of effects, target populations and uptake assumptions explored. N – number of sets of assumptions; Mid-value [low, high] – predicted population-level effectiveness corresponding to the mid [lowest, highest] parameter value (or middle of the interval when N = 2); F – female vaccination only; M+F or All – gender-neutral vaccination in a 2-sex and 1-sex model, respectively; L – lifelong; HSV-1 – HSV-1 negative; HSV-2+ – HSV-2 positive; (S, I, P) – susceptibility, infectivity, pathogenicity effects; *For Garnett – VE (39,73,88%) reflects the total reduction in symptoms incidence among vaccinated individuals resulting from the combined susceptibility (VE^S^_take_) and pathogenicity (VE^P^_take_) efficacies assumed. Garnett (S), (I, P), and (S, I, P) corresponds to scenarios Sc4, 1–3, and 5–6, respectively, as described in the text and Fig. 4; here the vaccine is effective among only HSV-1 negative women (only 40% of women). Upper and lower bounds in reductions represent uncertainty in the prediction based on variation of (i) vaccine efficacy only (Garnett), (ii) vaccine uptake only (Newton, Allsalaq), (iii) vaccine efficacy, duration of vaccine effects, and vaccine uptake (Freeman), and (iv) vaccine efficacy, duration of vaccine effects, vaccine uptake, and HSV natural history (Schwartz).

Fig. 3 and Supplemental Table S1 summarize model results for different sets of assumptions on vaccine characteristics, vaccination strategies, target population and uptake explored across models. These studies suggest that HSV-2 vaccination could reduce HSV-2 incidence rate by as little as 5% after 30 years or up to 88% after 10 years. Not surprisingly, studies that explored a wider range of assumptions and vaccine parameter values (Schwartz et al. (2005, 2007) [36], [37], Freeman et al. (2009) [38] produced wider ranges of predictions.

The earliest model by Newton et al. (2000) [33] optimistically predicted 62% and 81% reduction in HSV-2 incidence rate 10 years after routine vaccination of 1% and 2% of sexually-active adolescents monthly, achieving coverage of 43% and 61% after 10 years, respectively. They predicted even larger reduction (74% or 91% HSV-2 incidence reduction after 10 years) with a therapeutic-like mass vaccination scenario assuming that the infectivity of all HSV-2 infected individuals would be immediately reduced by 50% or 75%.

**Fig. 4:**
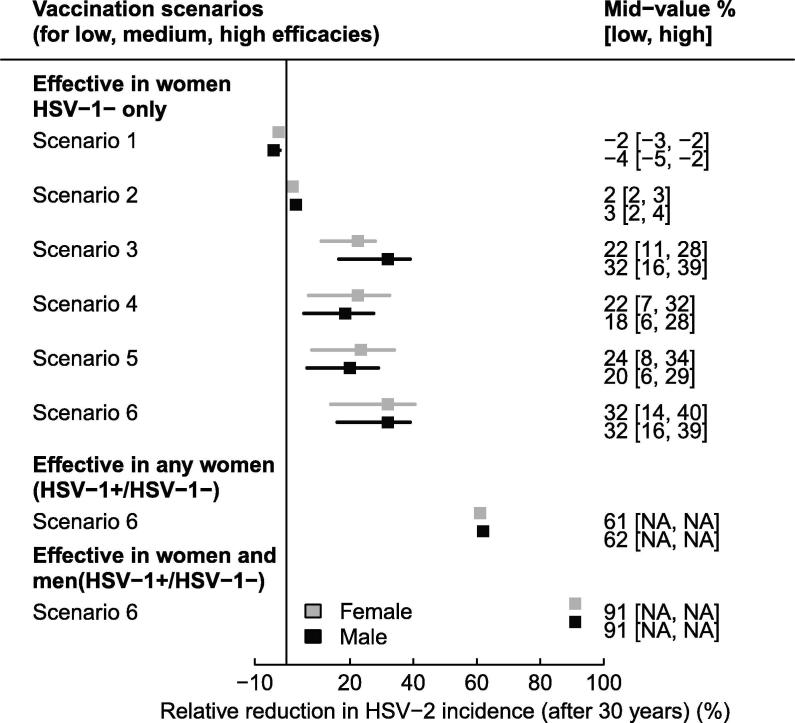
Predicted reduction in HSV-2 incidence rate after 30 years among males and females for the six scenarios presented in Garnett’s study where the vaccine is assumed to reduce: Sc1 – symptoms only (no reduction in infectivity), Sc2 – symptoms and symptomatic infectivity, Sc3 – symptoms and all (symptomatic and asymptomatic) infectivity, Sc4 – susceptibility only, Sc5 – susceptibility, symptoms and symptomatic infectivity, Sc6 – susceptibility, symptoms, and all infectivity among women HSV-1 negative only (only 40% of women), all women (not men), and both men and women. Other assumptions are as described in Fig. 3. Pos – positive, Neg – negative.

In Garnett et al. (2004) [35], the incidence reduction among women or men ranged between −5% and 40% 30 years after routine (50% of girls at sexual debut) and catch-up (5% adult women annually) vaccination across six scenarios and three efficacies (low, medium, high), where the vaccine was assumed to reduce: (Sc1) symptom frequency only (no reduction in infectivity), (Sc2) symptom frequency and symptomatic infectivity, (Sc3) symptom frequency and all infectivity (symptomatic and asymptomatic), (Sc4) susceptibility only, (Sc5) susceptibility, symptom frequency, and symptomatic shedding/infectivity, (Sc6) susceptibility, symptom frequency, and all shedding/infectivity (Fig. 3). Fig. 4 shows additional results by sex for scenarios Sc1-6. Interestingly, the population-level effectiveness was only marginally higher in women than men, even if vaccination was only effective in women, because men benefit from herd effects. Incidence reduction was larger among men only when they directly benefited from the reduction in infectivity of vaccinated women (Sc3). HSV-2 incidence reduction was higher for a vaccine with breakthrough and susceptibility effects (Sc5 or 6), than with susceptibility effects only (Sc4), especially when both symptomatic and asymptomatic infectivity were decreased (Sc6). In addition, the population-level effectiveness of a vaccine with infectivity (symptomatic and asymptomatic) and pathogenicity effects (Sc3) was larger than with susceptibility effects only (Sc4). Overall, incidence reductions were modest, despite being measured 30 years after vaccination began, primarily because the vaccine was assumed to only be effective among women uninfected with HSV-1 (assumed HSV-1 prevalence is 60%) (Fig. 4). In scenario 6, the population-level effectiveness nearly doubled when the vaccine was also effective among the 40% women who were HSV-1 infected and nearly tripled when assuming it was effective among 100% of men and women (Fig. 4). In scenario 1, only symptom frequency was affected by vaccination, leading to more frequent asymptomatic periods during which people were more sexually active causing vaccination to lead to a 5% increase in HSV-2 incidence (Fig. 4).

In two separate publications [36], [37], Schwartz et al., assessed population-level effectiveness of gender-neutral prophylactic and therapeutic vaccination varying several epidemiological factors (e.g., transmission rate, sexual lifespan, frequency of reactivation, duration of reactivation), vaccine characteristics (VE_take_, VE_S_, VE_I_, VE_R_, VE_L,_ duration of effects), and uptake simultaneously. Their earlier publication [36] predicted relatively modest overall reduction in cumulative HSV-2 incidence (3–36% over 10 years) following prophylactic vaccination considering that they explored high vaccine efficacies and uptake (Fig. 3). This is partly because they modeled routine vaccination of cohorts of new sexually-active cohorts without catch-up, and because they report a reduction in infection risk (i.e. cumulative incidence) rather than a reduction in incidence rate. In contrast, their later publication [37] predicted a larger reduction in the 10 year infection risk following therapeutic vaccination (24–80% over 10 years) despite exploring similar ranges of therapeutic effects and uptake to their first publication, and despite the lack of susceptibility effects. This is at least partly because routine vaccination targeted all HSV-2 infected individuals, resulting in a faster increase in coverage than routine vaccination of susceptibles. The predicted epidemiological impact with the therapeutic vaccine required that 30–90% of all asymptomatic HSV-2 positive people be vaccinated each year, which may be optimistic. Sensitivity analysis suggested that for a prophylactic vaccine the take (VE^S^_take_) the reduction in susceptibility (VE_S_), and coverage were more important than all other features varied; breakthrough effects had negligible impact. For a therapeutic vaccine it was more important to reduce infectivity (symptomatic and asymptomatic) than to reduce frequency and duration of reactivation episodes. Duration of sexual activity also influenced results, which may explain some variation across model predictions that represented different populations (e.g., younger versus older adults).

**Fig. 5:**
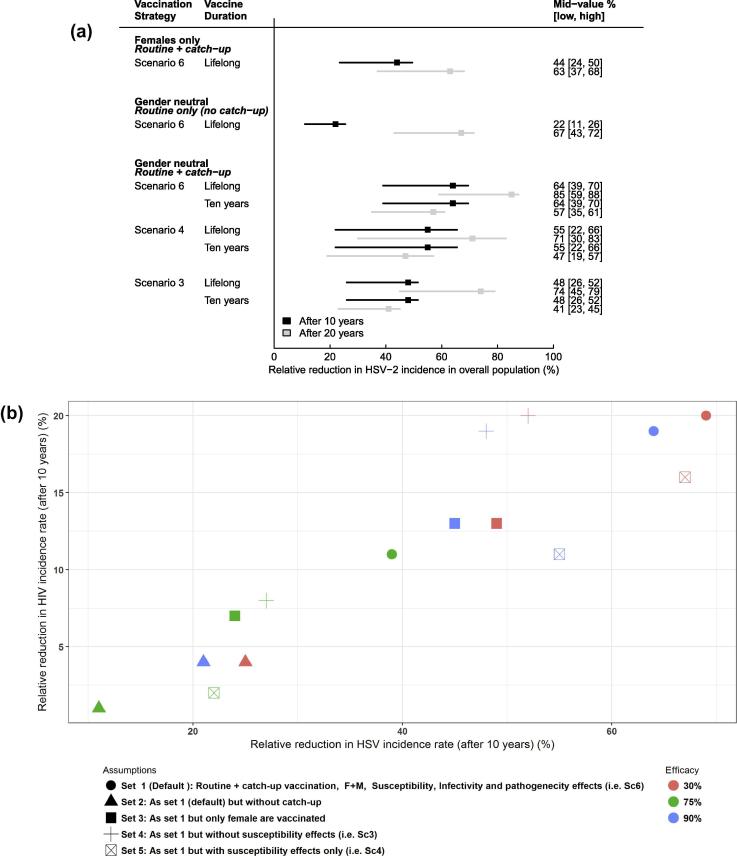
(A) Predicted reduction in overall HSV-2 incidence rate across selected sets of assumptions presented in Freeman et al. (2009). Vaccination strategies: routine vaccination at sexual debut with or without catch-up (50% uptake at 5 years), for a vaccine with different efficacies, duration, and effects (Sc3-6 defined in Table 1). (B) Scatter plot of the expected reduction in overall HIV incidence rate as a function of the overall reduction in HSV-2 incidence following HSV-2 vaccination for various sets of assumptions (vaccine effects and efficacies, duration of effects = 10 years) assuming 50% uptake. Vaccine effects: Sc4 – reduction in susceptibility only (VE_s_), Sc3 – reduction in shedding reactivation frequency and duration and all infectivity (VE_I_, VE_R_, VE_L_), Sc6 – both Sc3 and Sc4 (VEs, VE_I_, VE_R_, VE_L_). Mid [Low, High] values represent population-level effectiveness for efficacies of 75% [50%, 90%] as in Fig. 3.

Freeman et al. (2009) [38] predicted that prophylactic vaccination could reduce the HSV-2 incidence rate by 8% to 88% after 10 years of routine vaccination prior to sexual debut with catch-up among 15–29 year olds for various sets of assumptions varied across three dimensions: (1) vaccine effects: reduction in susceptibility only (as Garnett et al.’s Sc4), reduction in shedd reactivation frequency and duration and all infectivity (as Garnett et al.’s Sc3), or both (as Garnett et al.’s Sc6), (2) vaccine characteristics: efficacy, duration, and 3) vaccine uptake (Fig. 3). Freeman’s results for the prophylactic vaccine with breakthrough effects only (I,P) were similar to the results of Schwartz et al. (2007) [37] for the therapeutic vaccine (Fig. 3). Fig. 5A shows the influence of catch-up, gender-neutral or female-only vaccination, and duration of effects on model predictions, and Fig. S2 shows the incremental impact of these same features. The incremental impact of catch-up vaccination is substantial whereas that of gender neutral vaccination was weaker (Fig. S2). The incremental impact of a prophylactic vaccine with susceptibility and breakthrough effects was marginal compared to a vaccine with susceptibility effects only whereas vaccines with breakthrough or susceptibility effects only were similar (Fig. 5A, Fig. S2). In addition, assuming that HIV susceptibility and infectivity was higher during initial infection and reactivation episodes (25-fold, 10-fold per sex act), HIV incidence rates were indirectly reduced by 1 to 31% 10 years after vaccination across the ranges of assumptions explored. The reduction in overall HIV incidence was proportional to the reduction in overall HSV-2 incidence (Fig. 5B). However, their results did not include scale-up of HIV treatment.

Finally, Alsallaq et al. (2010) [39] suggested that HSV-2 prevalence could be reduced substantially by a vaccine that either reduces the frequency of reactivation episodes by 75% or reduces susceptibility by 75% plus reactivation frequency by 10% in high prevalence settings (results not shown). They predicted 21% and 30% HSV-2 cumulative incidence reductions 10 years after gender-neutral routine vaccination at sexual debut (100% uptake) and mass vaccination of 100% sexually active, respectively (VE_S_ = 30%, VE_R_ = 75%) (Fig. 3). Sensitivity analysis concluded that unless a prophylactic vaccine has low efficacy against infection, the incremental impact of reducing shedding frequency will be modest in the short-term, but would increase over time as more breakthrough infections occur.

## 5 Discussion

### 5.1 Summary and limitations

Eight modeling studies have been published to date on the potential impact of HSV-2 vaccination at a population level. These studies showed that even an imperfect prophylactic HSV-2 vaccine could have an important epidemiological impact on HSV-2 incidence, and could potentially impact HIV incidence in high prevalence settings as well. For example, in the one study that modeled both HSV-2 and HIV transmission in sub-Saharan Africa, a prophylactic vaccine with only 75% efficacy against infection could reduce HSV-2 incidence by more than 55% after 10 years, given 50% vaccination coverage of 14 year-olds and catch-up vaccination through age 29, and could also avert about 10% of incident HIV infections over the same period [38]. Therapeutic vaccines have been explored less extensively, and comparison with prophylactic vaccine models was difficult given differences in mechanisms, target populations, and delivery strategies, but available modeling work suggests these vaccines might also have public health benefits.

The available modeling studies have several limitations. First, most of the models were relatively simple and did not incorporate heterogeneities of specific settings and populations. Second, although one study considered the indirect effect of HSV-2 vaccination on HIV infection, none of the models included any other HSV outcomes in their measures of vaccine impact, such as neonatal herpes, the contribution of HSV-1 to morbidity including genital herpes, or psychosocial burden. Absent from all studies were costs, both for care and treatment and for vaccination, and thus any resulting cost-effectiveness analyses. Third, modeling work has been more limited for therapeutic vaccines, which may be the first HSV vaccine candidates approved since they are further along in clinical development [21], [22]. Therapeutic vaccines are an emerging concept in public health which have been studied little to date in terms of modeling; more research is warranted to fully understand their expected individual and population benefits, and to whom and how they should be targeted for optimal resource allocation. In addition, the intervention landscape has changed since these studies were published with scale-up of interventions like antiretroviral therapy (ART), pre-exposure prophylaxis (PrEP), and male circumcision for HIV infection, and potentially new antiviral therapies for HSV on the horizon [45]. No models of vaccine impact included any of these competing interventions, which, depending on the context, could affect vaccine impact.

Finally, studies varied in the degree to which they conducted appropriate sensitivity and uncertainty analyses, as well as how they presented results of vaccination impact; as point estimates only or with uncertainty ranges that reflected uncertainties in parameter assumptions and/or the ranges of scenarios explored. When comparing impact across models, we selected the lowest and highest values to reflect the ranges of scenarios explored in each publication. However, this means that we may have selected unrealistically wide bounds for vaccine impact, or conversely, given an unrealistically precise estimate of predicted vaccine impact. In addition, we selected predicted change in HSV-2 incidence in our model comparison as this was the most common outcome used by the studies for vaccine impact. However, achieving protection against new infection, whilst highly desirable of a vaccination program, is not necessarily the only way vaccines can be useful. Indeed, therapeutic vaccines could be primarily employed to reduce symptoms in those with symptomatic infection. This type of therapeutic vaccination program might have limited reach, depending on its effect on transmission, but might substantially improve the quality of life of individuals with diagnosed genital herpes and be cost-effective. Conversely, if it was deployed more widely among those HSV-2 positive, a therapeutic vaccine might have substantial impact on population incidence by decreasing transmission in serodiscordant partners. In this way modeling could help inform implementation decisions.

Despite these limitations, the evaluated studies yield several qualitative insights and provide a good foundation on which to build future modeling work. First, a vaccine does not necessarily need to have simultaneous effects on susceptibility, disease and infectiousness to be useful at the population level. Additional effects likely lead to increased impact, but this depends on relative efficacy: breakthrough effects of a prophylactic vaccine (i.e., reductions in infectivity among vaccinated individuals who experience breakthrough infection) are most important when vaccine susceptibility effects (i.e., efficacy against acquiring infection) are low. Second, catch-up vaccination appears important in maximizing vaccine impact. Third, in settings with relatively high HSV-1 prevalence, any vaccine needs to work in those already infected with HSV-1 to be useful at the population level, except for a prophylactic vaccine given to infants. Finally, even if a vaccine is only directly efficacious in one sex, it could still provide substantial herd immunity effects to the other sex given high enough levels of coverage and vaccine efficacy. In theory a prophylactic vaccine that works equally well in both sexes may not need to be used in both sexes to achieve public health goals; however, many other factors need to be considered including equity, which are beyond the scope of this review. Single-sex vaccination was explored in detail in prior models because an early vaccine trial showed efficacy only in HSV-1 seronegative women, which may not be relevant for future vaccines [19]. Overall, our findings show that factors related to vaccine deployment strategy (e.g., whether to employ catch-up vaccination) and biological features of the vaccine (e.g., whether the vaccine is efficacious regardless of HSV-1 status) are likely more important than whether the vaccine is efficacious for both sexes. These findings should be validated for a wider range of epidemiological patterns of infection and sexual behavior patterns.

### 5.2 Conclusions and future directions

Mathematical modeling of the potential impact of HSV-2 vaccination plays an important role in stimulating investment in the development of HSV vaccines and ensuring preparedness for vaccination programs before a vaccine comes to market. Once public health leaders have defined goals for HSV-2 prevention programs [22], mathematical models can help define the conditions in terms of vaccine cost and strategy likely to achieve these goals for vaccines with different characteristics, thereby informing vaccine development and introduction. Such models need to be able to generate relevant, dependable, and useful results for decision-makers. Critical to this will be cost-effectiveness analyses, which in turn require comprehensive, robust estimates of health utility and disability weights for HSV infection, which are currently lacking. Additional research is needed to provide these estimates. To fully capture the impact and cost-effectiveness of HSV vaccination, other disease outcomes directly or indirectly caused by HSV infection such as HSV-1 disease, neonatal herpes, and HIV, should be included in models.

Besides conducting cost-effectiveness analyses with a full range of outcomes, future models could be extended in three ways from those studied in this review, to make them more useful for catalyzing vaccine development and investment as well as informing future public health decision-making. First, they should incorporate greater complexity and heterogeneity, such as heterogeneous infection rates by age, sex and sexual activity, in order to resemble more closely transmission dynamics in countries. Heterogeneity in infection rates is an important determinant of intervention impact for STIs [46] and failure to accurately take such heterogeneities into account could lead to over- or underestimation of potential vaccine impact. For example, prior modeling work examining the impact of HSV-2 suppressive therapy on HIV transmission concluded that substantial HIV impact is possible with high coverage and long duration of suppressive therapy [47], but this depends on sexual mixing patterns and presence or absence of commercial sex work among other factors [48], [49]. However, with more detailed models come greater difficulties in mathematical tractability and specification, particularly for ordinary differential equation-based models, and these considerations must also be factored in. Further work to explore the dynamics and interactions between HSV-1, HSV-2 and HIV, the relationship between HSV shedding, viral load and infectivity, and the simplest way to model the natural history of HSV whilst retaining the key features important to vaccination impact will be critical in informing development of these more complex models [50].

Second, model exploration should have a stronger focus on sensitivity and uncertainty analysis. Future work should provide bounds around predictions that fully reflect parameter uncertainty, which are useful for policy makers in considering whether to make recommendations about vaccination or require more detailed data inputs. Models could also explore the multi-dimensional parameter space defined by epidemiological context (e.g., sexual behaviors, HSV-2 and HIV prevalence), vaccine characteristics (e.g., biological vaccine effects, duration of protection), and vaccination strategy (e.g., target population, coverage) in greater detail. Such an exploration could incorporate different epidemiological and country income-level scenarios as defined by the WHO expert consultation on HSV-2 vaccine impact modeling [32], to ensure models are relevant for global decision making. Using a common modeling framework, such sensitivity analyses could also rank characteristics in terms of their influence on incidence and incidence reductions in order to prioritize future data collection efforts.

Third, models should describe and study vaccine candidates and characteristics most relevant to the existing development pipeline and current public health needs, and should contain realistic vaccine deployment strategies mapped to the vaccine types and characteristics assessed. This is most notably absent for therapeutic vaccines, which are presently the furthest along in development with several candidates currently in, or having recently completed, phase I and II clinical trials (pipeline reviewed elsewhere [22]). Target populations and vaccine deployment strategies will differ markedly between prophylactic and therapeutic vaccine programs; care should be taken to simulate meaningful models of vaccine delivery, rather than those that are simple to model. For example, assuming that all individuals with HSV-2 infection, a largely undiagnosed infection, are instantaneously vaccinated with a therapeutic vaccine at 100% coverage [33], is clearly at odds with the realities of who would likely be the recipients of a therapeutic HSV-2 vaccine. The logistics of identifying and reaching suitable recipients represent practical considerations relevant to all vaccination programs that should be incorporated into future models of vaccine impact. Efforts are underway to define the preferred characteristics of HSV vaccines, such as vaccine indications, target populations, critical efficacy and safety considerations, and pertinent vaccination strategies, that would meet priority public health goals, particularly for low- and middle-income countries [22]. Future models should both incorporate and inform these preferred product characteristics to optimize their utility to advance development and implementation of HSV vaccines with global benefits.

HSV vaccines have the potential to alleviate a global disease burden, and vaccine development efforts are well underway [1], [22], [51]. Mathematical models take time to develop and fit; therefore modeling work should start before there is an approved HSV vaccine on the market. Models can be updated with efficacy data for the newest vaccine candidates as they progress through trials. Previous mathematical models have shown that substantial reductions in HSV-2 incidence are possible even with an imperfect vaccine if the right conditions are met. With the incorporation of the modeling considerations suggested here, substantial progress could be made toward understanding the future impact of an HSV-2 vaccine, which could improve the lives of millions of people worldwide.

## Conflicts/disclosures

The findings and conclusions of this study are those of the authors and do not necessarily represent the official positions of the Centers for Disease Control and Prevention, the World Health Organization, the UK National Health Service, the UK National Institute for Health Research, the UK Department of Health, or Public Health England.
