# Supplementary material for: Review of mathematical models of HSV-2 vaccination: Implications for vaccine development
Source: Vaccine. 2019 Nov 28;37(50):7396–407. doi: 10.1016/j.vaccine.2018.02.067 (PMC6892260; doi:10.1016/j.vaccine.2018.02.067)
Supplement: Supplementary data 1 [file mmc1.pdf]

Supplemental materials for 'Review of mathematical models of HSV-2 vaccination: Implications for vaccine development'

Newton model reproduction

Because Newton *et al*. (2000) only reported on reductions in prevalence associated with vaccination, and also because the model was fairly straight-forward to implement exactly as the authors had done, we reproduced their model in R using the deSolve package to solve the differential equations. The differential equations are:

$$\frac{dS}{dt} = -\mu S - \tau\kappa S - \beta S(\rho_H I_H + \rho_M I_M + \rho_L I_L)(1 - VE_I) + \mu N$$
$$\frac{dE}{dt} = -\mu E - \nu_1 E + \beta S(\rho_H I_H + \rho_M I_M + \rho_L I_L)(1 - VE_I)$$
$$\frac{dI_H}{dt} = -\mu I_H + \nu_1 E - \nu_2 I_H$$
$$\frac{dI_M}{dt} = -\mu I_M - \nu_4 I_M + \nu_3 I_L$$
$$\frac{dI_L}{dt} = -\mu I_L + \nu_4 I_M + \nu_2 I_H - \nu_3 I_L$$
$$\frac{dV}{dt} = -\mu V + \tau\kappa S$$

and the associated parameter symbols, descriptions, and values used in their manuscript and here are:

Symbol	Name	Value
μ	birth and death rate	1 / 9 years
ν_1	rate of transition from E to I_H	1 / 6 days
ν_2	rate of transition from I_H to I_L	1 / 12 days
ν_3	rate of transition from I_L to I_M	1 / 69 days
ν_4	rate of transition from I_M to I_L	1 / 4 days
ρ_H	probability of transmission from I_H	0.9
ρ_M	probability of transmission from I_M	0.5
ρ_L	probability of transmission from I_L	0.1
τ	proportion in whom the vaccine takes	1.0
κ	rate of vaccination	0, 1, or 2 % of susceptibles per month
β	contact rate	1.6 / year
VE_I	vaccine efficacy against infectiousness	0, 0.5, or 0.75

Under a prophylactic vaccine, Newton *et al.* (2000) assume sterilizing immunity vaccine effects, where susceptible individuals who are vaccinated enter a vaccinated compartment, and are no longer able to be infected. Under this scenario, κ takes a non-zero value, and determines the rate of vaccination.

Under a therapeutic vaccine, Newton *et al.* (2000) assume all currently and infected individuals have reduced infectivity, determined by VE_I.

Initial conditions are such that, from a total stable population size of 10000, 9500 are S, 1 is E, 2 are I_H, 470 are I_L, and 27 are I_R. Under either vaccination scenario, the model is ran from its initial conditions for 20 years before vaccination begins.

Vaccine impact is calculated by comparing the incidence rate in each vaccination scenario to the incidence rate in the null scenario 10 years after vaccination began.

Figure S1

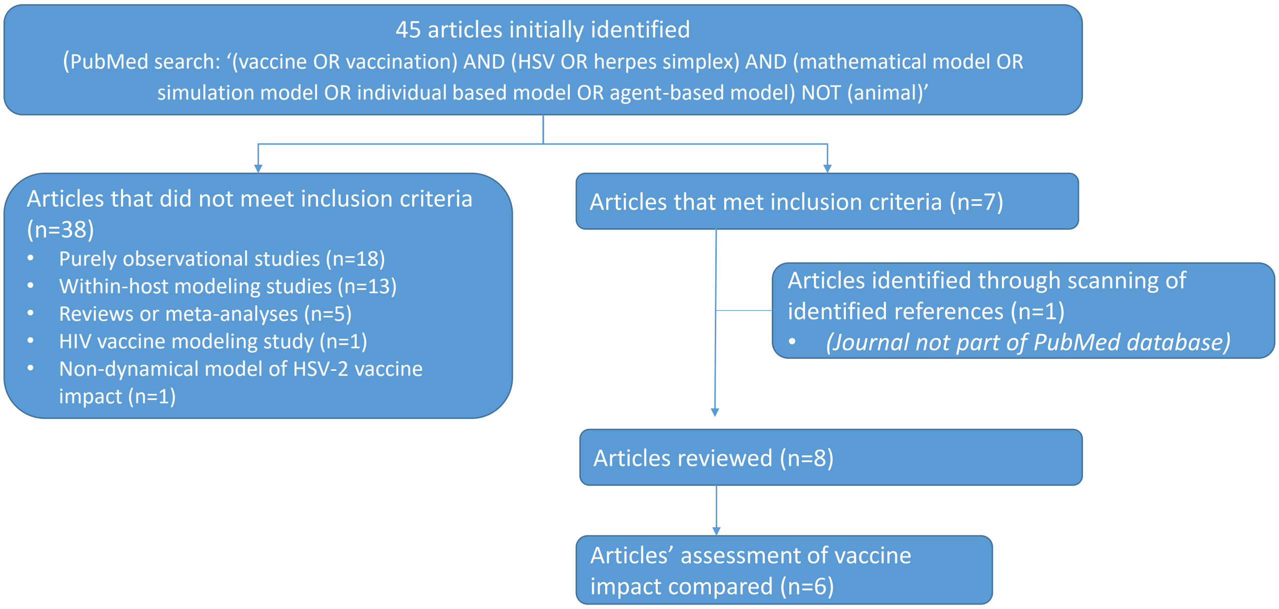
45 articles initially identified
(PubMed search: '(vaccine OR vaccination) AND (HSV OR herpes simplex) AND (mathematical model OR simulation model OR individual based model OR agent-based model) NOT (animal)'
Articles that did not meet inclusion criteria (n=38)
• Purely observational studies (n=18)
• Within-host modeling studies (n=13)
• Reviews or meta-analyses (n=5)
• HIV vaccine modeling study (n=1)
• Non-dynamical model of HSV-2 vaccine impact (n=1)
Articles that met inclusion criteria (n=7)
Articles identified through scanning of identified references (n=1)
• (Journal not part of PubMed database)
Articles reviewed (n=8)
Articles' assessment of vaccine impact compared (n=6)

Figure S2

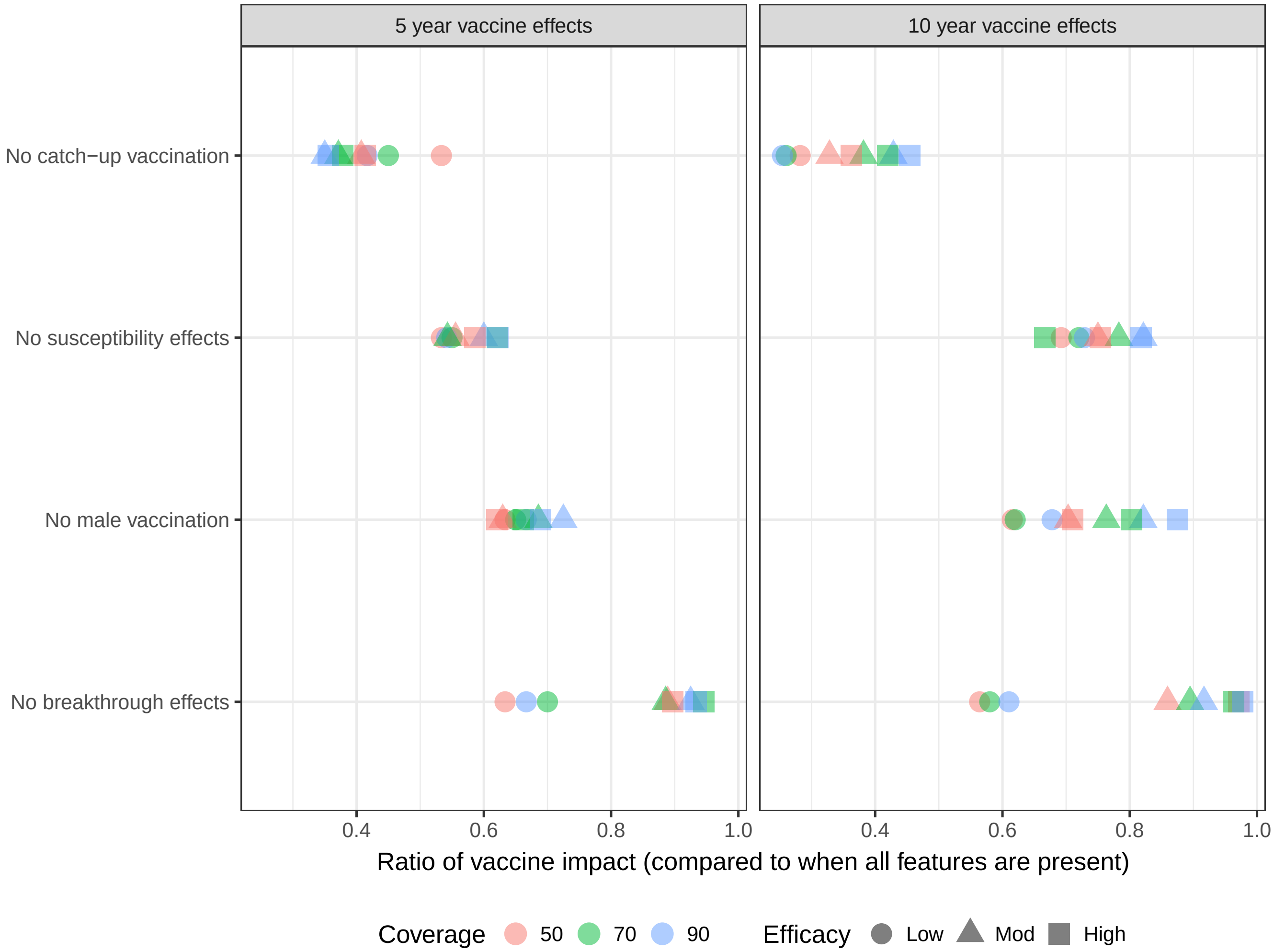

Comparison of incremental vaccine impact in Freeman *et al.* when a single feature is not present 10 years after vaccination began. Vaccine impact is defined as the incidence rate reduction 10 years after vaccination began. Scenarios defined along the y-axis are compared to a base-scenario that had the greatest vaccine impact in which all four features (i.e. catch-up vaccination, susceptibility effects, vaccination of both sexes, and breakthrough effects) are present. Each ratio is compared assuming the same level of coverage (color in the figure), vaccine efficacy (shape), and duration of conferred vaccine effects (left versus right panel). Lack of catch-up vaccination has the strongest detrimental effect, since vaccine impact is about 60% lower without it.

Table S1

Author	Model framework	Sexual activity	Setting and baseline prevalence	Vaccine efficacy	Vaccine take	Vaccine duration	Target population for vaccination	Vaccination strategy and coverage	Predicted HSV-2 incidence reduction
Prophylactic vaccines - susceptibility effects only									
Garnett (S)	2-sex deterministic compartmental	Hetero-geneous levels	North America HSV-2: 25% HSV-1: 60%	VE_S=100%	13 or 62%	Life	HSV-1 negative females primarily. Examined males+females in a single scenario (not shown)	50% coverage at sexual debut and 5% of sexually active unvaccinated suscpetibles per year	5-33% after 30 years
Freeman (S)	2-sex stochastic discrete individual based model	Hetero-geneous levels	Sub-Saharan Africa HSV-2: 15 or 41% HIV: 3 or 19%	VE_S=30, 70, or 90%	100%	5 years, 10 years or life	Both sexes aged 15-29 years old regardless of HSV-1 status. Examined scenarios with only females in Fig. 5	Routine vaccination at sexual debut, and initial catch-up vaccination in order to achieve 50, 70 or 90% coverage after 5 years.	10-86% after 10 years
Newton (S)	1-sex deterministic compartmental	Homo-geneous levels	Unspecified geographic setting HSV-2: 38%	VE_S=100%	100%	Life	1-sex model without taking into account HSV-1 status.	1 or 2% of sexually active susceptibles vaccinated per month	62-81% 10 years
Prophylactic vaccines - susceptibility and breakthrough effects									
Schwartz 2005 (S,I,P)	1-sex deterministic compartmental	Homo-geneous levels	North America HSV-2: 22 or 60%	Varied independently: VE_S=VE_I= VE_L=VE_R= 30-100%	Varied: 30-100%	Varied: 10-20 years	1-sex model without taking into account HSV-1 status.	30-90% varied coverage at sexual debut	3-36% after 10 years
Alsallaq (S,I)	1-sex deterministic compartmental	Hetero-geneous levels	Sub-Saharan Africa HSV-2: 52%	VE_S=30% VE_R=75%	100%	Life	1-sex model without taking into account HSV-1 status.	Mass vaccination with 100% coverage at sexual debut and mass vaccination with 100% coverage of adults within one year	21-30% after 10 years
Garnett (S,I,P)	2-sex deterministic compartmental	Hetero-geneous levels	North America HSV-2: 25% HSV-1: 60%	VE_S=VE_I= 100%	13 or 62% for S 39 or 88% for I	Life	HSV-1 negative females primarily. Examined males+females in a single scenario (not shown)	50% coverage at sexual debut and 5% of sexually active unvaccinated suscpetibles per year	15-40% after 30 years
Freeman (S,I,P)	2-sex stochastic discrete individual based model	Hetero-geneous levels	Sub-Saharan Africa HSV-2: 15 or 41% HIV: 3 or 19%	VE_S=VE_L= VE_R=30, 70, or 90%	100%	5 years, 10 years or life	Both sexes aged 15-29 years old regardless of HSV-1 status. Examined scenarios with only females in Fig. 5	Routine vaccination at sexual debut, and initial catch-up vaccination in order to achieve 50, 70 or 90% coverage after 5 years.	15-88% after 10 years
Prophylactic vaccines - breakthrough effects only									
Garnett (I,P)	2-sex deterministic compartmental	Hetero-geneous levels	North America HSV-2: 25% HSV-1: 60%	VE_P=100%	39 or 88%	Life	HSV-1 negative females primarily. Examined males+females in a single scenario (not shown)	50% coverage at sexual debut and 5% of sexually active unvaccinated suscpetibles per year	11-37% after 30 years
Freeman (I,P)	2-sex stochastic discrete individual based model	Hetero-geneous levels	Sub-Saharan Africa HSV-2: 15 or 41% HIV: 3 or 19%	VE_L=VE_R= 30, 70,or 90%	100%	5 years, 10 years or life	Both sexes aged 15-29 years old regardless of HSV-1 status. Examined scenarios with only females in Fig. 5	Routine vaccination at sexual debut, and initial catch-up vaccination in order to achieve 50, 70 or 90% coverage after 5 years.	8-72% after 10 years

Therapeutic vaccines - no susceptibility effects									
Schwartz 2007 (I,P)	1-sex deterministic compartmental	Homo-geneous levels	North America HSV-2: 22%	Varied independently: VE_I= VE_L=VE_R= 30-100%	Varied: 30-100%	Varied: 10-20 years	1-sex model without taking into account HSV-1 status.	30-90% of those with latent HSV-2 infection vaccinated per year	24-80% after 10 years
Newton (I)	1-sex deterministic compartmental	Homo-geneous levels	Unspecified geographic setting HSV-2: 38%	VE_I=50 or 75%	100%	Life	1-sex model without taking into account HSV-1 status.	Mass vaccination of all current and newly HSV-2 infected	74-91% 10 years
Excluded from impact comparison									
Lou (S)	2-sex deterministic compartmental	Homo-geneous levels	North America HSV-2: 22%	VE_S=100%	Varied: 0-100%	Life	HSV-1 negative females only	Varied 0-100% coverage at sexual debut	0-100% after 1000 years
Podder (S,I,P)	1-sex deterministic compartmental	Homo-geneous levels	Unspecified geographic setting HSV-2: arbitrarily small	VE_S=60% VE_I=30-40% VE_L=33%	60%	15 years	1-sex model without taking into account HSV-1 status.	50% coverage at sexual debut and 60% of sexually active unvaccinated susceptibles per year	-

Table 1: Characteristics and predictions of HSV-2 vaccine modeling literature scenarios. Biological vaccine effects in parentheses reflect vaccines with some combination of susceptibility (**S**), infectiousness (**I**), and pathogenicity (**P**) effects. Multiple scenarios per article are possible when an article considered different scenarios defined by the type(s) of biological vaccine effects being conferred. Articles are organized by vaccine design (prophylactic, prophylactic with breakthrough effects, and therapeutic) and within these categories by increasing magnitude of impact. 1-sex models do not differentiate between males and females; 2-sex models represent females and males separately, allowing for vaccination of one or both sexes. Bounds represent upper and lower predictions either based on ranges from all the samples generated (Schwartz), or upper and lower estimates when only two data points were predicted (Garnett, Alsallaq, Freeman, Newton). *Vaccine increased duration of symptomatic period compared to unvaccinated
